# Young adults with a history of depression and/or anxiety: the role of sociodemographic and health-related factors in not being sickness absent

**DOI:** 10.1093/joccuh/uiaf077

**Published:** 2025-12-26

**Authors:** Jurgita Narusyte, Iman Alaie, Annina Ropponen, Mo Wang, Pia Svedberg

**Affiliations:** Division of Insurance Medicine, Department of Clinical Neuroscience, Karolinska Institutet, Stockholm, Sweden; Division of Insurance Medicine, Department of Clinical Neuroscience, Karolinska Institutet, Stockholm, Sweden; Division of Clinical Psychology, Department of Psychology, Uppsala University, Uppsala, Sweden; Division of Insurance Medicine, Department of Clinical Neuroscience, Karolinska Institutet, Stockholm, Sweden; Division of Workability and Working Careers, Finnish Institute of Occupational Health, Helsinki, Finland; Division of Insurance Medicine, Department of Clinical Neuroscience, Karolinska Institutet, Stockholm, Sweden; Division of Insurance Medicine, Department of Clinical Neuroscience, Karolinska Institutet, Stockholm, Sweden

**Keywords:** young adults, sick leave, mental health, employment sector

## Abstract

**Objectives:**

The continuity of mental health problems from childhood to adulthood is well acknowledged, as is the impact on work ability. However, knowledge is scarce about individuals who maintain work ability and have no sickness absence (SA), despite mental health problems. The aim was to identify sociodemographic and health-related factors among private and public employees with a history of depression and/or anxiety, and no SA.

**Methods:**

This prospective cohort study included 9039 Swedish twin individuals born between 1975 and 1986, with and without a history of depression and/or anxiety, and employed in the private or public sectors. Survey data from 2005 were used to classify self-rated depression, anxiety, and overall health. Data on SA, education, occupational class, outpatient health care use, and prescribed antidepressants were obtained from national registries. Participants were prospectively followed for SA from 2006 to 2020. Logistic regression analyses were applied to calculate odds ratios (ORs) with 95% CIs.

**Results:**

Approximately 37% of individuals with previous depression and/or anxiety were not on SA during follow-up, compared with 54% of those without such history. Lower use of antidepressants implied higher odds for no SA among both private (OR: 2.09; 95% CI, 1.64-2.66) and public (OR: 2.38; 95% CI, 1.78-3.19) employees with previous depression and/or anxiety. Having fewer visits to outpatient health care was significantly associated with no SA (ORs: 2.22-3.60). Being a white-collar worker implied higher odds for no SA only among those privately employed (OR: 1.39; 95% CI, 1.10-1.76).

**Conclusions:**

Primarily, health-related factors seemed to play a role in no SA among young employees with previous depression and/or anxiety.

## Teaser

Key Points

- Mental health problems in early life increase the risk of future sickness absence. However, knowledge is scarce on what factors contribute to maintaining work ability among young adults with previous mental health problems.

- Several health-related, but not sociodemographic, factors tend to be important for maintaining work ability among both privately and publicly employed individuals with a history of depression and/or anxiety.

- Future research of work ability among individuals with mental health problems needs to consider health-related factors.

## 1. Introduction

Mental health problems among young adults incur a major health burden^1^ as well as increased risk for long-term health- and work-related consequences, including sickness absence (SA).^2^^-^^5^ Marginalization from working life due to SA has been identified as a societal challenge in several European countries, including Sweden.^6^ Despite the documented increased risk for SA among young adults experiencing mental health problems early in life,^7^^-^^9^ some individuals tend to remain in paid work. Research is scarce regarding factors specific to individuals who retain their work capacity despite prior mental health problems, yet such information is highly relevant when considering SA prevention strategies.

Work may contribute to better mental health, both in the general population as well as for individuals with mental health problems.^10^ A wide range of factors, including sociodemographic, health-related, and work-related, have been studied to identify those promoting participation in paid work and return to work among individuals with mental health problems.^11,12^ The proposed mechanisms supporting individuals to remain in paid work involve factors related to organizational climate, social support, job demands/control, coping strategies, health, personal context, and interventions.^11^ However, specific knowledge regarding the importance of sociodemographic and health-related factors in different employment sectors and occupational classes (ie, white-collar vs blue-collar) is still insufficient. Such knowledge may be vital to better understand and tailor strategies aiming to promote work participation for individuals with mental health problems.

Higher rates of SA have been observed among employees in the public sector compared with the private employment sector.^13^^-^^15^ A recent study of young adults reported that public sector employees had a higher risk of future SA due to common mental disorders compared with private sector employees.^16^ However, in both employment sectors, an early onset of depression or anxiety symptoms may imply an increased risk of SA of similar magnitude.^17^ Thus, it seems that individuals with early mental health problems tend to possess a vulnerability to SA, which may be robust to differences between employment sectors. It is probably not surprising given that, for example, depressive symptoms often include loss of interest or energy as well as cognitive and concentration difficulties.^18^ The symptoms are similar regardless of the employment sector. However, it is unclear whether the employment sector and occupational class are important for stable work participation (ie, with no SA) among young individuals with mental health problems.

Data for twins offer a unique opportunity to control for confounding arising from unmeasured familial factors including genetics and shared (mainly childhood) environment. Genetic factors were previously reported to explain approximately 30%-40% of the variance in depression/anxiety and SA, as well as occupational status.^19^^-^^21^ This suggests that the epidemiological analyses of these conditions could benefit considerably from accounting for such familial factors, in order to avoid misinterpreting possible nonfamilial environmental influences.

The aim of this study was, by combining survey and register data, to identify sociodemographic and health-related factors among privately and publicly employed young adults with and without a history of depression and/or anxiety but no future occurrence of SA during follow-up. We also aimed to examine the influence of twin resemblance (zygosity) on these associations.

## 2. Methods

### 2.1. Study sample

The study sample was drawn from the prospective longitudinal population-based study, the Swedish Twin project Of Disability pension and Sickness absence (STODS), whose participants were born between 1975 and 1986 (*n* = 12 725). The twins were invited by the Swedish Twin Registry (STR) to participate in 2 different surveys and the data were included in STODS.

Twins born during 1975-1986 were among the participants in the cross-sectional survey, the Study of Twin Adults: Genes and Environment (STAGE), in 2005,^22^ when the twins were aged 20-30 years (*n* = 8609). STAGE included extensive batteries of questions regarding physical and mental health, lifestyle, and sociodemographic characteristics. In the present study, those individuals with responses on depression and/or anxiety symptoms were included (*n* = 7716).

Twins born in 1985-1986 participated in the Twin study of Child and Adolescent Development (TCHAD) (*n* = 2845), which was a longitudinal study of physical and mental health spanning from childhood to adulthood.^23^ The twins and their parents were contacted at 4 waves of measurement. In the present study, the twins’ responses on depression and/or anxiety symptoms in 2005, at ages 19-20 years, were used (*n* = 1668).

**Figure 1:**
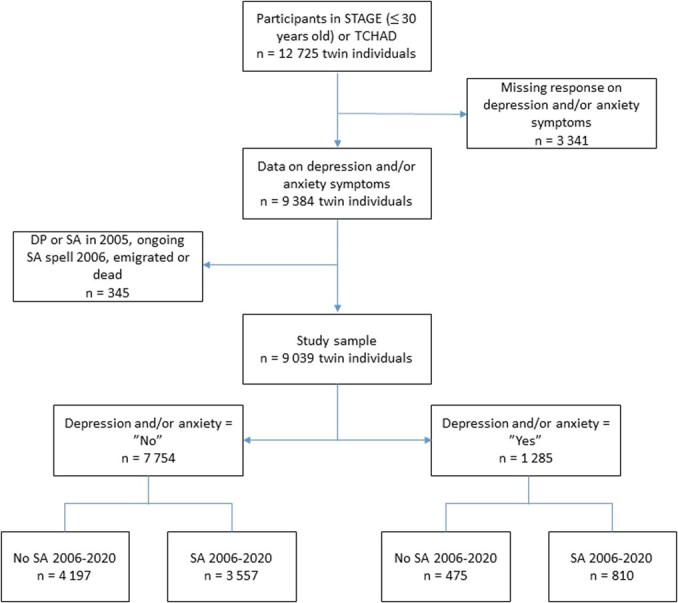
Flow-chart of the study sample selection. STAGE: The Study of Twin Adults: Genes and Environment; TCHAD: Twin study of Child and Adolescent Development; SA: sickness absence; DP: disability pension.

All twins who participated in TCHAD or STAGE were followed annually from the year 2006 until the year 2020 regarding the occurrence of SA. Those who were granted disability pension prior to 2006, had ongoing SA spells in 2006, or died or emigrated during follow-up, were excluded from the sample (*n* = 345). The final analytic sample included 9039 twin individuals, of which there were 1285 twins with a history of depression and/or anxiety and 7754 twins with no such history. The selection of the study sample is shown in Figure 1.

### 2.2. Ethical approval

The study was approved by the Regional Ethical Review Board of Stockholm (DNRs 2007/524-31; 2010/1346-32/5; 2014/311-32; 2015/1809-32; 2017/128-32) and the Swedish Ethical Review Authority (DNRs 2021-03482, 2022-03878-02). The study followed the principles embodied in the Declaration of Helsinki.

### 2.3. Measures

#### 2.3.1. Depression and anxiety

In TCHAD, depression and anxiety were assessed by the Internalizing Problems Subscale in the Adult Self-Report.^24^ In the present study, the presence of clinically relevant depression/anxiety was denoted by a commonly accepted and validated cut-off of T-scores of 65 or above.^21^ In STAGE, the lifetime history of major depressive and of generalized anxiety disorders was assessed using the computerized Composite International Diagnostic Interview–Short Form (CIDI-SF), which was adapted from its original design for 12-month prevalence to assess lifetime prevalence of *Diagnostic and Statistical Manual IV* (DSM-IV) disorders.^25^ The Internalizing scale has been repeatedly shown to correspond well with DSM-IV–based assessment of anxiety and mood disorders (eg, reference^26^). Thus, a common dichotomous variable was created to denote presence of depression and/or anxiety (yes/no) in TCHAD or STAGE, and individuals with the score alternative “yes” were included in further analyses (*n* = 1285).

#### 2.3.2. Sociodemographic factors

Family status (1 = married or cohabiting, 2 = single), education level (high = more than 12 years, low = less or equal to 12 years) in 2020 as well as employment sector (1 = private, 2 = public) and occupational class (1 = blue-collar, 2 = white-collar) were derived from data obtained from the Longitudinal Integrated Database for Health Insurance and Labour Market Studies (LISA) database^27^ administered by Statistics Sweden.

Private sector included individuals employed by private companies, whereas public sector covered individuals employed by the state, counties, and municipalities. Individuals were classified as employed in the public or private sectors after being employed in either sector for at least 3 years in total during the follow-up. Similarly, individuals were classified as having white-collar or blue-collar occupations. If an individual was employed in both employment sectors and occupational classes for an equal number of years, the sector and class during the last years of follow-up were chosen.

#### 2.3.3. Health-related factors

Self-rated health was assessed using participants’ responses to the question “How do you rate your general health?” in TCHAD and STAGE. In this study, the responses “Excellent,” “Good” and “Moderate” were collapsed into 1 = Good health, whereas “Fairly poor” and “Poor” were collapsed into 2 = Poor health.

Data on specialized outpatient health care visits in 2006-2020 were obtained from the Swedish National Patient Registry. Specialized outpatient health care use during follow-up was measured in 3 ways: (1) at least 1 visit, (2) mean and (3) median number of visits. The total number of visits during follow-up was further categorized into 3 categories: 0 = No visits, 1 = 1-3 visits, 2 = 4-8 visits, and 3 = 9 or more visits. The diagnoses related to pregnancy and delivery were excluded from the analyses (International Classification of Diseases 10 [ICD-10]: O00-O99).

Use of prescribed antidepressants (Anatomical Therapeutic Chemical [ATC] code N06A) in 2006-2020 was measured by using data from the Prescribed Drug Registry. Two categories were created: No = No prescribed antidepressants or a single prescription, and Yes = Antidepressants were prescribed at least twice.

#### 2.3.4. Sickness absence

Data on SA spells due to any diagnosis were obtained from the Micro-Data for Analysis of the Social Insurance System (MiDAS) database at the Swedish Social Insurance Agency. In Sweden, all people aged 16-65 years and who have income from work or unemployment benefits are entitled to sickness benefits in case of temporarily reduced working capacity due to disease or injury. The sickness benefit covers up to 80% of the individual’s lost income and is paid by the employer for the first 14 days of the SA spell; an SA spell lasting longer than 14 days continues to be paid by the Swedish Social Insurance Agency. Thus, the data on SA retrieved from MiDAS include only those SA spells that exceeded 14 days.

For the analyses, a dichotomous variable was created to measure the nonoccurrence of SA (yes/no) during follow-up 2006-2020. An individual was assumed to have no SA if no incident SA spells were registered during follow-up. To ensure the temporality of the studied association, only incident SA spells were considered in the analyses; that is, the SA spells that were continuing from 2005 (baseline) to 2006 were excluded.

### 2.4. Statistical analyses

Descriptive statistics, including frequencies and proportions of health-related and sociodemographic factors, were calculated separately for participants with and without previous depression and/or anxiety. Differences between groups were tested using the chi-squared test. Further, we conducted logistic regression analyses to estimate the association between health-related and sociodemographic factors and no SA during follow-up, adjusting for intrapair twin dependency. The analyses were stratified by previous history of depression and/or anxiety as well as for employment sector. Crude odds ratios (ORs) with 95% CIs were calculated (Model 1) as well as adjusted ORs for sex, education (Model 2), and zygosity of the twins (Model 3). We also conducted a sensitivity analysis where nonincident SA spells were considered when evaluating the nonoccurrence of SA; that is, SA spells that were ongoing at the start of the follow-up year 2006 were included in the analysis. All statistical analyses were performed using SAS software version 9.4.^28^

## 3. Results

Descriptive statistics of the study sample are presented in Table 1. Most study participants were women, with a higher proportion among those with (75%) than without (54%) a previous history of depression and/or anxiety. The proportion of individuals with an education of 12 years or longer was higher among those without previous depression and/or anxiety (55%) as compared with those with such a history (51%). The proportion of those working in the private sector was higher among study participants without previous depression and/or anxiety (60%) than those with previous depression and/or anxiety (49%). More than half of the participants without previous depression and/or anxiety (54%) did not have any incident SA spell during follow-up as compared with 37% with such a history.

**Table 1:** Descriptive statistics of the study sample (*n* = 9039).

	**Previous depression and/or anxiety (*n* = 1285)**		**No previous depression and/or anxiety (*n* = 7754)**		***P* value** ^**a**^
	***n***	**%**	***n***	**%**	
**Women**	968	75	4190	54	<.0001
**Mean age (SD) at baseline 2005**	24 (3.4)		24 (3.6)		
**Educational attainment**					.003
** ≤12 years**	642	51	2828	55	
** >12 years**	621	49	3424	45	
**Missing information**	22	2	77	1	
**Zygosity**					.33
**Monozygotic (MZ)**	541	42	3369	43	
**Dizygotic (DZ, same-sex and opposite sex)**	703	55	4201	54	
**Missing**	41	3	184	2	
**Employment sector**					<.0001
**Private**	627	49	4668	60	
**Public**	549	43	2749	35	
**Missing information**	109	8	337	4	
**SA 2006-2020**					<.0001
**At least 1 incident SA spell**	810	63	3557	46	
**No SA spells**	475	37	4197	54	

Abbreviation: SA, sickness absence.

^a^ *P* value was calculated excluding categories with missing information and refers to differences between individuals with and without previous depression and/or anxiety.

Bold values denote statistical significance.

Among those privately employed, the proportion of women was higher among those with previous depression and/or anxiety (55%) compared with those with no such symptoms (34%) (Table 2). Among the study participants with a previous history of such symptoms, 68% had higher education, 96% had rated their health as good, and 28% had received prescription antidepressant medication at some point, all factors significantly different from those without a history of the symptoms. The proportions of individuals with blue-collar occupations or having at least 1 visit to specialized outpatient health care were not significantly different between those with and without a history of depression and/or anxiety. For employees in the public sector, no significant differences between those with and without a history of depression and/or anxiety were observed for education, occupational class, or visits to specialized outpatient health care. Approximately 28% of those with previous depression and/or anxiety had received a prescription of antidepressants, and 96% rated their health as “good,” both of which were significantly different from those with no history of depression and/or anxiety.

**Table 2:** Health-related and sociodemographic factors among private and public employees with and without a history of depression/anxiety and no sickness absence (SA) spells during follow-up 2006-2020 (*n* = 4672).

	**Previous depression and/or anxiety**	**No previous depression and/or anxiety**	***P* value**
	**Private sector**		
	***n* = 234**	***n* = 2677**	
**Women**	128 (55)	913 (34)	<.0001
**Education >12 years**	154 (68)	1607 (61)	.04
**Occupational class blue-collar**	104 (45)	1129 (43)	.54
**Married or cohabiting**	80 (35)	1219 (47)	.001
**Self-rated health good**	225 (96)	2650 (99)	<.001
**Granted disability pension 2006-2020**	<5 (1)	<5 (0.1)	.01
**Use of antidepressants 2006-2020**	65 (28)	154 (6)	<.0001
**At least 1 visit to specialized outpatient care 2006-2020**	207 (88)	2307 (86)	.33
	**Public sector**		
	***n* = 163**	***n* = 1256**	
**Women**	122 (75)	749 (60)	<.001
**Education >12 years**	135 (84)	1011 (82)	.47
**Occupational class blue-collar**	52 (32)	321 (26)	.08
**Married or cohabiting**	63 (39)	563 (46)	.13
**Self-rated health good**	156 (96)	1242 (99)	.001
**Granted disability pension 2006-2020**	<5 (1)	<5 (1)	.09
**Use of antidepressants 2006-2020**	46 (28)	92 (7)	<.0001
**At least 1 visit to specialized outpatient care 2006-2020**	150 (92)	1120 (89)	.26

All values are *n* (%).

The number of outpatient health care visits was significantly different between those with and without previous history of depression/anxiety (Table 3). The average and median number of visits were significantly higher among those with previous depression and/or anxiety, as compared with those without these symptoms, in both private and public sectors. In both sectors, approximately 35%-42% of the individuals with a history of depression and/or anxiety had more than 8 visits whereas among those without such history, 25%-27% of the individuals had more than 8 visits to outpatient health care during follow-up.

**Table 3:** Outpatient health care visits among private and public employees with and without a history of depression/anxiety among those with no sickness absence (SA) spells during follow-up 2006-2020.

	**Previous depression and/or anxiety**	**No previous depression and/or anxiety**	***P* value**
	**Private sector**		
	***n* = 234**	***n* = 2677**	
**Number of visits to outpatient health care 2006-2020**			.004
**0**	27 (12)	370 (14)	
**1-3**	57 (24)	864 (32)	
**4-8**	69 (29)	779 (29)	
**>8**	81 (35)	664 (25)	
**Mean number of visits to outpatient health care 2006-2020**	10	8	.003
**Median number of visits to outpatient health care 2006-2020**	6	5	.01
	**Public sector**		
	***n* = 163**	***n* = 1256**	
**Number of visits to outpatient health care 2006-2020**			.001
**0**	13 (8)	136 (11)	
**1-3**	39 (24)	382 (30)	
**4-8**	42 (26)	393 (31)	
**>8**	69 (42)	345 (27)	
**Mean number of visits to outpatient health care 2006-2020**	11	8	<.0001
**Median number of visits to outpatient health care 2006-2020**	8	5	.05

All values are *n* (%) or *n*.

In Table 4, the ORs of no SA during follow-up among individuals with and without previous depression and/or anxiety are presented. For private sector employees with previous depression and/or anxiety, the adjusted odds of no SA were higher when having a white-collar occupation (OR 1.42; 95% CI, 1.13-1.79), not being prescribed antidepressants (OR 2.15; 95% CI, 1.69-2.73), and having fewer than 9 visits to outpatient health care (ORs 2.22-3.09). In contrast, for public sector employees with previous depression and/or anxiety, the association between white-collar occupation and no SA was not statistically significant and neither was the association between good self-rated health and no SA. The absence of prescribed antidepressants implied higher odds of no SA (OR 2.47; 95% CI, 1.85-3.30) as well as having fewer than 9 visits to outpatient health care (ORs 2.24-3.60). For both private and public employees with no previous depression and/or anxiety, significant associations were observed between white-collar occupation, absence of prescribed antidepressants, and having fewer than 9 visits to outpatient health care. In all analyses, the results changed only slightly when adjusting for twins’ zygosity (Model 3).

**Table 4:** Odds ratios (ORs) for no sickness absence during 2006-2020 among private and public employees with and without previous depression and/or anxiety.

	**Private sector (*n* = 5183)**		
	**OR (95 % CI)**		
	**Model 1**	**Model 2**	**Model 3**
**Previous depression and/or anxiety *(n = 675)***			
**White-collar (blue-collar = ref)**	1.46 (1.19-1.78)	1.42 (1.13-1.79)	1.39 (1.10-1.76)
**Good self-rated health (poor health = ref)**	1.76 (0.99-3.12)	1.84 (1.01-3.37)	1.85 (0.96-3.54)
**No use of antidepressants (use = ref)**	2.31 (1.82-2.93)	2.15 (1.69-2.73)	2.09 (1.64-2.66)
**Number of outpatient health care visits (>8 = ref)**			
**0**	3.52 (2.71-4.56)	3.09 (2.35-4.06)	3.18 (2.45-4.14)
**1-3**	2.94 (2.30-3.78)	2.78 (2.16-3.57)	2.82 (2.19-3.63)
**1-3**	2.36 (1.83-3.04)	2.22 (1.72-2.86)	2.22 (1.70-2.89)
**No previous depression and/or anxiety *(n = 4508)***			
**White-collar (blue-collar = ref)**	1.31 (1.24-1.38)	1.26 (1.19-1.34)	1.26 (1.18-1.34)
**Good self-rated health (poor health = ref)**	1.28 (0.92-1.78)	1.19 (0.86-1.63)	1.21 (0.87-1.68)
**No use of antidepressants (use = ref)**	2.53 (2.20-2.89)	2.34 (2.04-2.67)	2.32 (2.03-2.66)
**Number of outpatient health care visits (>8 = ref)**			
**0**	2.52 (2.35-2.70)	2.24 (2.08-2.41)	2.24 (2.08-2.42)
**1-3**	2.15 (2.01-2.31)	2.01 (1.88-2.16)	2.02 (1.88-2.17)
**4-8**	1.68 (1.56-1.81)	1.64 (1.52-1.76)	1.64 (1.52-1.77)
	**Public sector (*n* = 3292)**		
	**OR (95 % CI)**		
	**Model 1**	**Model 2** ^a^	**Model 3** ^b^
**Previous depression and/or anxiety *(n = 548)***			
**White-collar (blue-collar = ref)**	1.12 (0.85-1.47)	1.07 (0.79-1.45)	1.04 (0.76-1.41)
**Good self-rated health (poor health = ref)**	1.47 (0.76-2.87)	1.58 (0.82-3.07)	1.53 (0.79-2.95)
**No use of antidepressants (use = ref)**	2.61 (1.95-3.49)	2.47 (1.85-3.30)	2.38 (1.78-3.19)
**Number of outpatient health care visits (>8 = ref)**			
**0**	4.11 (2.93-5.75)	3.60 (2.53-5.12)	-
**1-3**	3.11 (2.32-4.16)	2.83 (2.09-3.84)	-
**4-8**	2.32 (1.70-3.16)	2.24 (1.64-3.05)	-
**No previous depression and/or anxiety *(n = 2744)***			
**White-collar (blue-collar = ref)**	1.38 (1.25-1.53)	1.39 (1.25-1.55)	1.39 (1.25-1.54)
**Good self-rated health (poor health = ref)**	1.47 (0.90-2.40)	1.42 (0.92-2.19)	1.41 (0.91-2.19)
**No use of antidepressants (use = ref)**	3.02 (2.51-3.64)	2.73 (2.28-3.27)	2.72 (2.27-3.28)
**Number of outpatient health care visits (>8 = ref)**			
**0**	3.05 (2.72-3.42)	2.53 (2.26-2.84)	2.56 (2.27-2.88)
**1-3**	2.45 (2.20-2.72)	2.20 (1.98-2.46)	2.22 (1.99-2.48)
**4-8**	1.88 (1.68-2.11)	1.82 (1.62-2.03)	1.81 (1.62-2.03)

Model 1: crude estimates. Model 2: estimates adjusted for sex and educational level. Model 3: estimates adjusted for sex, educational level, and twin zygosity.

^a^The analyses were only adjusted for sex due to nonconvergence.

^b^No convergence.

The results of the sensitivity analyses differed only marginally from the OR estimates calculated in the main analyses (Table 4) and therefore are not shown.

## 4. Discussion

In this longitudinal cohort study of 9039 young twin adults with and without a history of depression and/or anxiety, we aimed to examine characteristics relevant to individuals who do not experience medically certified SA spells (>14 days) throughout early-to-middle adulthood.

Compared with the twins with no history of depression and/or anxiety, a lower proportion of individuals with no SA seemed to have good self-rated health. Further, a higher proportion of those with a history of depression and/or anxiety had been prescribed antidepressants as well as making more visits to specialized outpatient health care. The results suggest that although young adults with a history of depression and/or anxiety symptoms seem to maintain their work capacity and have no SA during the following years, they still seem to experience a long-term impact of these mental health problems as indicated by the use of antidepressants and specialized outpatient health care. Our findings are in line with well-acknowledged long-lasting adverse consequences of mental health problems experienced in early years (eg,^2^^-^^3^). In addition, the results of our study extend the existing knowledge by showing that this association seems also to hold for individuals who do not have any interruptions in their working life in terms of SA.

In contrast to health-related factors, individuals with previous depression and/or anxiety and maintained working capacity seem not to differ regarding sociodemographic factors from individuals without any history of these mental health problems. Among those with no SA during follow-up, the proportions of individuals with high educational levels as well as blue-collar occupations were similar despite previous depression and/or anxiety. Neither was the proportion of those married or cohabiting significantly different between those with and without previous depression and/or anxiety, among the publicly but not privately employed. These findings align with previous studies, where low education, blue-collar occupation, and being single were shown to increase risk for future SA.^15,29,30^ Our results add to the previous findings by showing that the association does not differ depending on the experience of depression and/or anxiety in early adulthood.

The results were only slightly different between those employed in the private and public sectors. The association between having a white-collar occupation and no SA during follow-up was significant for those with previous depression and/or anxiety employed in the private, but not the public sector. The absence of prescribed antidepressants and fewer visits to outpatient health care implied higher odds of no SA among employees in both sectors. Also, in both sectors, all estimates changed marginally when adjusting for twins’ zygosity, suggesting that familial factors (ie, genetics and early family environment) tend to play a minor role in the studied associations. A few disparities between the employment sectors in our results are unexpected, as higher SA rates in the public than the private sector have been acknowledged previously.^13^^-^^15^ However, the higher SA rates were suggested to be primarily attributable to differences in sex, age, health status, or size of the company rather than differences between the employment sectors.^14,31^ Also, a recent report based on the same data as the present study showed that experiencing depression/anxiety in early years implied risk for future SA of a similar extent in both private and public sectors.^17^ Thus, our findings in the present study align well with previous research and suggest that the differences in SA between the employment sectors tend to become negligible when factors related to morbidity and work are considered.

We found no statistically significant association between self-rated health and no SA among individuals with or without previous depression and/or anxiety. This finding differs from previous research, according to which self-rated health is a well-acknowledged predictor of morbidity and mortality^32,33^ as well as SA.^34^ Good self-rated health was also reported to be one of the factors facilitating stay and performance at work among individuals with mental health problems.^11^ The results from the present study may, however, be interpreted considering potential sickness presenteeism. That is, private sector employees with previous depression and/or anxiety may tend to keep working despite suboptimal health.^35^ Sickness presenteeism was previously reported to be more common among manual workers compared with nonmanual workers, as well as among those with temporary compared with permanent employment contracts.^36,37^ Since approximately half of the study participants had blue-collar occupations, and the fact that temporary employment is much more common at young ages, sickness presenteeism may also be a relevant issue among young adults experiencing mental health problems. However, the role of sickness presenteeism could not be investigated in the present study, yet it is important to consider in future studies.

Young employees in the public sector with no SA were more likely to visit outpatient health care compared with those in the private sector. That is, among those with no SA, the odds of not having any contact with outpatient health care were lower among the publicly compared with the privately employed. This is an interesting finding as previous studies reported higher levels of SA in the public than the private sector, the difference usually attributable to employees’ health.^16,31^ Potentially, our results may suggest that employees in the public sector seek health care to a higher degree at early stages of symptom or disease progression, which, in turn, may prevent future SA. Mindful that the majority of the public sector employees in this study had high education, this interpretation is well in line with previous results showing that individuals with higher socioeconomic status tend to show higher utilization of specialized health care services.^38^

This study has several strengths, the first of these being a population-based and longitudinal study with long follow-up. Another strength relates to the data used. We had a unique possibility to combine survey and register data, which enabled us to investigate self-reported information on depression/anxiety, including mild and undiagnosed symptoms, in combination with register data on SA. Use of register data additionally implies no loss to follow-up as well as no information or recall biases.

Several limitations also need to be mentioned. First, since the study sample was relatively small, the studied associations need to be replicated in studies using larger samples. For the same reason, the importance of familial factors could only be tested in brief (ie, by adjusting for twin similarity) and not using the framework of discordant twins, due to the low numbers of twin pairs discordant for SA. Nevertheless, the findings of this twin study are generalizable to the population at large, as twins are representative of the general population.^39^ Related to this, the generalization of the study results may be limited for cohorts of older ages as well as for countries other than Nordic countries due to different social insurance systems. Further, data on SA spells shorter than 14 days are not available in national registries in Sweden; thus, the occurrence of short SA spells could not be investigated. Another limitation may be related to the classification of the employment sector and occupational class. The categorization of both measures was based on the total number of years of being employed in each of the sectors or occupational classes during the study follow-up. That is, possible switches between employment sectors and occupational classes during a particular year could not be considered. A further limitation relates to the use of two different measures of depression/anxiety in this study. Despite the previously documented good agreement between the child behavior checklist (CBCL) Internalizing scale and DSM-IV–based assessment of depression/anxiety,^26^ some cases of depression/anxiety may have been misclassified, which in turn could bias the results toward the null. It is also important to note that both measures of depression/anxiety in our study were based on self-reports. Since self-reported assessments can help to capture individuals with undiagnosed conditions or those experiencing less severe symptoms, they may offer a more accurate reflection of the true prevalence of depression/anxiety in the general population. However, this may also lead to somewhat inflated association estimates compared with those based solely on clinically diagnosed cases. In addition, although the prescription of antidepressants in this study was primarily used as a proxy for the treatment of mental health problems (since we had no information on other types of treatments in our data), it is important to note that antidepressants may be a part of the treatment of other medical conditions as well, including, for example, pain conditions. Finally, although the analyses were adjusted or stratified for several factors of importance for SA, the issue of residual confounding remains, as we could not adjust for several work- and health-related factors (eg, psychosocial work environment or cognitive functioning) that were previously reported to influence SA.^30,40^

## 5. Conclusions

In both private and public sectors, employees with a history of depression and/or anxiety and no future SA seem to differ in regards to health-related, but not sociodemographic, factors as compared with individuals without these mental health problems.

## Data Availability

The data are not publicly available due to the legal restrictions set out in the General Data Protection Regulation, the Swedish law SFS 2018:218, the Swedish Data Protection Act, the Swedish Ethical Review Act, and the Public Access to Information and Secrecy Act. These types of sensitive data can only be made available after legal review, for researchers who meet the criteria for access to these types of sensitive and confidential data. The data used in this study are administered by the Division of Insurance Medicine, Karolinska Institutet. Readers may contact stods-cns@ki.se regarding the data.
